# Investigation on public stigma and empathy ability of undergraduate nursing students towards schizophrenia

**DOI:** 10.3389/fmed.2026.1944425

**Published:** 2026-09-29

**Authors:** Luyu Zhang, Xueqin Zhang, Zhenling Lu

**Affiliations:** 1 Central Sterile Supply Department, Shandong Provincial Hospital, Jinan, China; 2 School of Nursing, Shandong First Medical University, Tai'an, China

**Keywords:** empathic capacity, nursing education, public stigma, schizophrenia, undergraduate nursing students

## Abstract

**Objective:**

This study explores the current status of public stigma and empathy ability of undergraduate nursing students towards schizophrenia, and analyzing the relationship between empathy and public stigma, providing a theoretical basis for schools to take relevant measures to improve the empathy ability of undergraduate nursing students.

**Methods:**

This study employed a cross-sectional design and, through using convenience sampling method to select undergraduate nursing students from the first to the fourth year of a certain medical university's School of Nursing as the survey subjects, and used the Schizophrenia Public Stigma and Empathy Ability Questionnaire for the investigation.

**Results:**

The average score of public stigma towards schizophrenia among 300 undergraduate nursing students was (3.78 ± 1.03) points, and the empathy ability score was (3.75 ± 1.47) points; there was a negative correlation between the empathy ability of nursing students and the total score of public stigma towards schizophrenia (*P* < 0.01); In multiple linear regression analysis, empathy remains independently and negatively correlated with public stigma (*P* < 0.01).

**Conclusion:**

The perspective taking, compassion and concern, and perspective shifting of empathy ability are the main influencing factors of public stigma towards schizophrenia among undergraduate nursing students. Medical colleges and universities should take relevant measures to cultivate and improve the empathy ability of undergraduate nursing students to improve the quality of care for patients with schizophrenia.

## 1 Introduction

Schizophrenia (SZ) is a group of mental disorders whose etiology has not yet been fully elucidated. It typically onset in young and middle-aged adults and is often characterized by disturbances in perception, thought, emotion, and behavior (1). Schizophrenia accounts for 70% of mental illness cases in China, with a relapse rate of approximately 80% within five years, which adversely affects patients’ quality of life (2). Societal prejudice and discrimination against people with schizophrenia have a lasting negative impact on them and affect their treatment adherence (3). Studies have shown that factors such as the fear of relapse in schizophrenia are associated with public stigma (4); public stigma refers to the negative attitudes and perceptions held by the general public toward certain individuals, groups, or phenomena. Nurses are the most important group in direct contact with patients with schizophrenia, and their attitudes influence patients’ psychological well-being and perceptions (5, 6). Empathy is the ability to put oneself in another's shoes, observe and experience the patient's emotions, and provide feedback accordingly. Research has shown that empathy influences the level of public stigma toward schizophrenia (7). Nurses’ capacity for empathy is crucial for establishing positive nurse-patient relationships, promoting the stabilization of psychiatric patients’ conditions, and advancing the field of nursing (8). Undergraduate nursing students in China represent a vital pool of future nursing talent. Therefore, fostering a constructive attitude toward patients with mental illness during their training holds significant educational importance. International research on the public stigma surrounding mental illness is relatively extensive. In mainland China, a review study found that public awareness of schizophrenia remains low, mental health literacy is inadequate, and more than 50% of people with schizophrenia experience long-term stigma associated with their condition (9). Chinese studies have also documented public stigma among psychiatric nurses and low levels of empathy (10, 11); however, there are relatively few studies examining the association between public stigma toward schizophrenia and empathy among nursing students (12). Therefore, the present cross-sectional study aimed to (1) describe the levels of public stigma toward schizophrenia and empathy among undergraduate nursing students; (2) examine the association between empathy and public stigma; and (3) examine whether empathy remains independently associated with public stigma after accounting for psychiatry-related learning, clinical internship, and family history of mental illness. We hypothesized that higher empathy would be associated with lower public stigma. The findings of this study are intended to provide guidance for schools in implementing measures to cultivate and enhance the empathy skills of undergraduate nursing students, as well as in designing nursing curricula.

## 2 Subjects and methods

### 2.1 Study population and research design

This study employs a cross-sectional questionnaire survey design. Undergraduate nursing students at a medical university in Shandong Province were selected for a questionnaire survey using convenience sampling. Inclusion criteria: (1) full-time undergraduate nursing students; (2) students who provided informed consent to participate in this study. Exclusion criteria: (1) midwifery students; (2) nursing students who had transferred from associate's to bachelor's degree programs; (3) students who had transferred from other majors to the nursing program.

For sample size calculation, *a priori* calculations were performed using G Power 3.1 software for multiple linear regression (four independent variables, two-tailed *α* = 0.05, power = 0.80, and a medium effect size of 0.15), resulting in a minimum sample size of 85 participants. To account for invalid questionnaires, a total of 320 questionnaires were distributed. Of the 320 distributed questionnaires, 20 were excluded due to incomplete entries or invalid response patterns (e.g., identical answers to all questions). Ultimately, 300 valid questionnaires were analyzed (a response rate of 93.8%). As shown in Figure 1.

**Figure 1:**
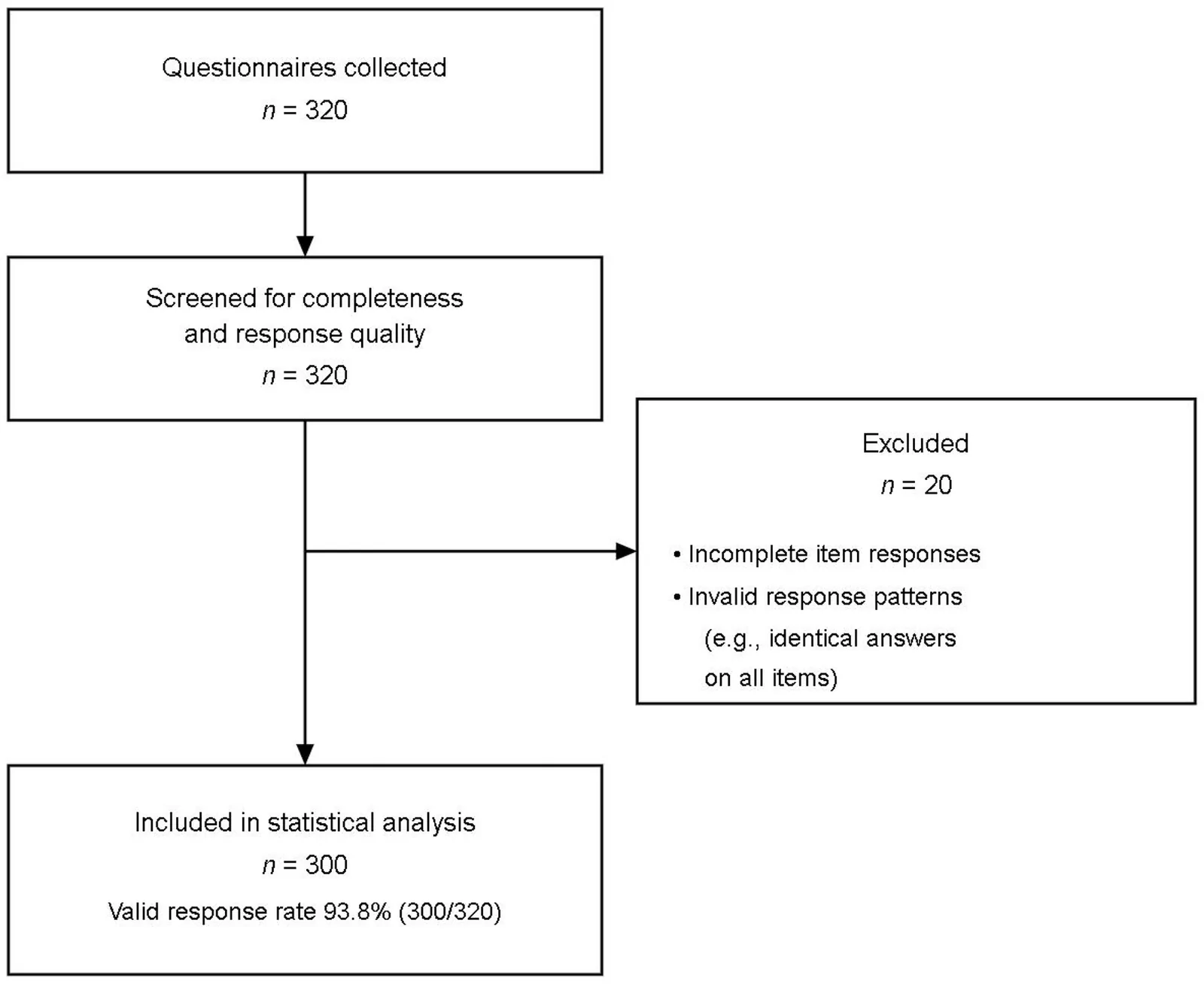
Flow diagram of questionnaire screening and inclusion.

### 2.2 Ethical considerations

This study was approved by the Ethics Committee of Shandong First Medical University (Approval No. R202511190517). The study was conducted in strict accordance with the Declaration of Helsinki. All participants were informed of the study's purpose, the anonymity of their responses, and their right to withdraw from the study at any time; written or implied informed consent was obtained prior to completing the questionnaire. No personally identifiable information was collected.

### 2.3 Research tools

The questionnaire consists of three parts: (1) A general information questionnaire designed based on a literature review and expert consultation: gender, grade level, ethnicity, household registration, parents’ educational attainment, family history of mental illness, clinical internship experience, and whether students have studied psychiatry-related content. (2) The Public Stigma Scale for Mental Illness (PSSMI) (13). Developed by Winnie W.S. Mak et al. from the Department of Psychology at The Chinese University of Hong Kong, there are a total of 21 items, including the Stigma Subscale and the Acceptance Subscale. A 6-point Likert scale is used; higher scores on the scales indicate that nursing students exhibit more severe stigmatizing attitudes and less acceptance toward people with mental illness, the Chinese version used in this study has previously been administered to nursing students in China. In the sample of this study (14), the Cronbach's *α* coefficient is 0.89. (3)The JSE-S (15) measures nursing students’ empathy and consists of 20 items divided into three dimensions: perspective-taking, compassion, and empathy. It uses a 7-point Likert scale; higher scores indicate greater empathy among nursing students, this study used the Chinese version compiled and validated by An Xiuqin (16) et al. In the sample of this study, the Cronbach's *α* coefficient is 0.90.

The questionnaire was administered anonymously and distributed and collected on-site. The researchers explained the purpose of the survey and provided instructions for completing it to the participants.

### 2.4 Statistical methods

Data were analyzed using SPSS 22.0 statistical software. Quantitative and categorical data are presented as $\bar{X}\pm S$ and *n* (%) respectively. Independent-samples t-tests and one-way analysis of variance (ANOVA) were used to compare two or more groups; Pearson's correlation analysis was used to examine the relationship between undergraduate nursing students’ empathy and public stigma toward schizophrenia; A multiple linear regression analysis was conducted to identify independent factors influencing nursing students’ perceptions of public stigma toward schizophrenia, including the adjusted R^2^, confidence intervals, and information regarding assumptions and multicollinearity. A *p*-value < 0.05 indicated a statistically significant difference.

## 3 Results

### 3.1 Demographic characteristics of the study participants

A total of 300 valid questionnaires were collected for this study. The demographic characteristics of the participants are presented in Table 1.

**Table 1:** Demographic characteristics of survey participants (*n* = 300).

Item	Group	Frequency (*n*)	Percentage (%)
Gender	Male	75	25.00
	Female	225	75.00
Grade	Freshman	69	23.00
	Sophomore Year	90	30.00
	Junior Yea	70	23.33
	Senior	71	23.67
Ethnicity	Han	270	90.00
	Other Ethnicities	30	10.00
Household registration type	Urban	111	37.00
	Rural	189	63.00
Father's educational attainment	Elementary school or below	30	10.00
	Junior high school	150	50.00
	High school	71	23.67
	College	49	16.33
Mother's educational attainment	Elementary school or below	70	23.33
	Junior high school	145	48.33
	High school	43	14.33
	College	42	14.00
Are there any family members with a mental illness?	Yes	38	12.67
	No	262	87.33
Have you completed a clinical internship?	Yes	163	54.33
	No	137	45.67
Have you studied psychiatry-related topics?	Yes	210	70.00
	No	90	30.00

### 3.2 Scores on dimensions of public stigma toward schizophrenia and empathy among undergraduate nursing students

Undergraduate nursing students scored an average of (3.78 ± 1.03) on public stigma toward schizophrenia and (3.75 ± 1.47) on empathy. The specific scores for each dimension are shown in Table 2.

**Table 2:** Scores on dimensions of public stigma toward schizophrenia and empathy among undergraduate nursing students.

Project	Dimensions and total score	Number of entries	$\bar{X}\pm S$
Public stigma	Stigma Scale	12	3.75 ± 1.32
	Acceptance Scale	9	3.83 ± 1.12
	Total Score	21	3.78 ± 1.03
Empathy	Perspective-Taking	10	3.41 ± 1.69
	Compassion	8	3.77 ± 1.91
	Putting Yourself in Someone Else's Shoes	2	3.57 ± 2.10
	Total Score	20	3.75 ± 1.47

### 3.3 Univariate analysis of nursing students’ perceptions of public stigma toward schizophrenia

Psychiatry-related educational background, family history of mental illness, and clinical internships are associated with scores on the public stigma scale (*P* < 0.05). there were no statistically significant differences in the scores for gender, grade level, ethnicity, household registration type, father's educational attainment, and mother's educational attainment (*P* > 0.05), as shown in Table 3.

**Table 3:** Univariate analysis (*n* = 300).

Project	Category	Number of people (*n*)	$\bar{x}\pm s$	*t/F*	*P*
Gender	Male	75	3.86 ± 0.91	0.720	0.472
	Female	225	3.76 ± 1.07		
Grade	Freshman	69	3.76 ± 1.12	2.091	0.101
	Sophomore	90	4.00 ± 1.09		
	Junior	70	3.64 ± 0.98		
	Senior	71	3.66 ± 0.88		
Ethnicity	Han	270	3.78 ± 1.05	0.437	0.662
	Other Ethnicities	30	3.70 ± 0.94		
Residence status	Urban	111	3.77 ± 1.02	−0.125	0.900
	Rural	189	3.79 ± 1.05		
Father's educational level	Elementary school or below	30	3.85 ± 1.17	1.537	0.205
	Junior high school	150	3.75 ± 1.03		
	High school	71	3.97 ± 1.07		
	College	49	3.57 ± 0.87		
Mother's educational level	Elementary school or below	70	3.86 ± 1.04	0.695	0.556
	Junior high school	145	3.82 ± 1.09		
	High school	43	3.69 ± 0.90		
	College	42	3.61 ± 0.98		
Are there any family members with a mental illness?	Yes	38	3.33 ± 0.93	−2.979	0.003*
	No	262	3.85 ± 1.03		
Have you completed a clinical internship?	Yes	163	3.55 ± 0.93	−4.320	0.000*
	No	137	4.06 ± 1.09		
Have you studied psychiatry-related subjects?	Yes	210	3.64 ± 0.94	−3.841	0.000*
	No	90	4.13 ± 1.15		

* indicates *P* < 0.01.

### 3.4 Analysis of the correlation between nursing students’ empathy and public stigma toward schizophrenia

The results show that there is a significant negative correlation between nursing students’ empathy and the total score for public stigma toward schizophrenia, with a correlation coefficient of −0.659 (*P* < 0.01). Perspective-taking, compassion, and empathy all showed significant negative correlations with the total public stigma score (*P* < 0.01); see Table 4 for details.

**Table 4:** Correlation analysis (*r*).

Item	Total public stigma score	Total empathy score	Viewpoint selection	Compassion and care	Perspective-taking
Total public stigma score	1				
Total empathy score	−0.659	1			
Viewpoint selection	−0.440	0.766	1		
Compassion and care	−0.502	0.718	0.362	1	
Perspective-taking	−0.643	0.628	0.376	0.432	1

Note: All *P* < 0.001.

### 3.5 Multifactorial analysis of public stigma toward schizophrenia among undergraduate nursing students

Using the total score of public stigma toward schizophrenia among undergraduate nursing students as the dependent variable, this study examined family history of mental illness, clinical internships, psychiatry-related coursework, and empathy as independent variables. The coding of the independent variables is shown in Table 5; there is an independent negative correlation between empathy and public stigma, as shown in Table 6. In the multivariate model, family history, clinical internships, and psychiatry-related coursework were all non-significant. The adjusted R^2^ values from the regression analysis, diagnostics for multicollinearity, and regression hypothesis tests are presented in Tables 7–9.

**Table 5:** Independent variable coding table.

Factor	Variable description	Coding
X1	Whether there is a family member with a mental illness	Yes=1, No = 2
X2	Whether the student has completed a clinical internship	Yes=1, No = 2
X3	Whether the student has studied psychiatry-related knowledge	Yes=1, No = 2
X4	Empathy	Substitute raw values

**Table 6:** Multiple linear regression analysis (*n* = 300).

Variable	*B*	*SE*	*β*	*t*-value	*P*	95%CI
Constants	4.621	0.332		13.920	<0.001	3.968,5.274
Total empathy score	−0.440	0.032	−0.624	−13.953	<0.001	−0.503,−0.377
Are there any family members with mental illness?	0.256	0.136	0.082	1.883	0.061	−0.012,0.524
Have you completed a clinical internship?	0.141	0.105	0.068	1.340	0.181	−0.066,0.348
Have you studied psychiatry-related topics?	0.100	0.114	0.044	0.876	0.382	−0.124,0.324

*R*^2^ = 0.451, *F* = 60.667, *P* < 0.001.

**Table 7:** Goodness of fit.

*R*	*R*2	Adjust *R*2	*F*	*df*	*P*
0.672	0.451	0.444	60.684	4,295	<0.001

**Table 8:** Multicollinearity diagnosis.

Independent variables	Tolerance	VIF	Determination
Total empathy score	0.929	1.076	No significant multicollinearity
Whether any family members have a mental illness	0.974	1.026	No significant multicollinearity
Whether the participant has completed a clinical internship	0.721	1.388	No significant multicollinearity
Whether the participant has studied psychiatry-related topics	0.731	1.369	No significant multicollinearity

**Table 9:** Hypothesis testing for regression.

Assumption	Statistics	*P*/Value	Conclusion
The residuals are approximately normally distributed	Shapiro–Wilk W = 0.992; Jarque–Bera=4.286; Skewness 0.064, excess kurtosis −0.571	0.093/0.117	Do Not Reject the Normal Distribution (*α*=0.05)
Independent observation	Durbin-Watson	0.858	DW = 0.858, which is less than 1.5, suggesting a positive correlation among residuals under the current sorting method; cross-sectional surveys are typically interpreted as independent samples, but it is not advisable to emphasize the independence of time series.
Homogeneity of residual variances	Breusch-Pagan LM = 24.165	<0.001	Rejects the assumption of homoscedasticity; heteroscedasticity is present
Linear setting	Ramsey RESET(for the quadratic term in the fitted values)F = 16.539	<0.001	This may indicate the presence of nonlinearity.
Multicollinearity	All Independent Variables VIF	1.388	No significant multicollinearity

## 4 Discussion

### 4.1 Analysis of nursing students’ perceptions of public stigma toward schizophrenia and their level of empathy

In this study, undergraduate nursing students scored relatively high on the overall measure of public stigma toward schizophrenia, indicating that such stigma is widespread among nursing students. These findings are consistent with those of Chinese scholars Wu Yuexi (6) and Wu Hao (10), and international scholar Chen (17), among others, regarding public stigma among psychiatric nurses. This result suggests that this may be due to long-standing societal stereotypes and prejudices toward people with schizophrenia, consistent with the findings of Zhou Qian (18) et al. Although nursing students receive education in relevant humanities courses during their academic training, most have not yet had direct contact with individuals with schizophrenia. They believe that psychiatric nurses, due to their prolonged interaction with patients, are inevitably subjected to frequent verbal and physical aggression and bear significant physical and psychological stress (19). Furthermore, prejudices held by their friends and family toward individuals with schizophrenia indirectly influence nursing students’ career choices and their attitudes and perceptions toward psychiatric patients, resulting in a high level of stigma toward individuals with schizophrenia. Empathy is a crucial skill for promoting nurse-patient communication and enhancing cognitive abilities, and it can effectively facilitate an individual's emotional growth (20). Research indicates (21) that empathy plays a vital role in the nurse-patient relationship; nursing students with high levels of empathy can put themselves in the patients’ shoes, provide appropriate care, and alleviate patients’ physical and psychological suffering. This study reveals that undergraduate nursing students’ empathy scores are at a slightly below-average level, consistent with the findings of Qu Fangge (22) and Sun Liangliang (23). This suggests that, although nursing students recognize the potential role of empathy in addressing public stigma toward people with schizophrenia in clinical settings, the perceived effectiveness of empathy in their actual work and daily lives remains less than ideal. Research has indicated (24) that empathy influences nursing students’ professional attitudes, and that the empathy levels of undergraduate nursing students require further improvement. A possible explanation is that, although undergraduate nursing students have taken certain humanities and social science courses that foster a professional ethos of respect for patients, their academic curriculum places greater emphasis on foundational professional knowledge, with humanities courses constituting a relatively small proportion of their studies. Therefore, greater attention should be paid to the development of empathy in nursing education to provide effective guidance for enhancing nursing students’ empathy skills.

### 4.2 The link between empathy and public stigma

The results of this study show that undergraduate nursing students’ public stigma toward schizophrenia is negatively correlated with their empathy, with the findings of Hu Zhengying et al. (5). Research indicates that there is a correlation between medical students’ attitudes and behaviors toward patients with mental illness and their capacity for empathy. It has also been found that whether medical students have received specialized training in psychiatry is related to their level of empathy and their attitudes toward patients (25). The findings suggest that college students’ attitudes toward mental illness vary depending on their academic year and whether they have taken relevant courses; educational activities targeting mental illness, if scientifically planned, appear to bring about positive changes in these attitudes (26). Furthermore, research has found that empathy is one of the core elements in maintaining a positive doctor-patient relationship between medical students and patients. Cultivating empathy within the curriculum can effectively enhance students’ levels of empathy (27). It is worth noting that by putting oneself in another's shoes, this approach encourages people to focus on the patient's experience of the illness, thereby helping to provide attentive and compassionate care (28); psychiatry courses and clinical rotations may exert their effects in part by increasing knowledge and structured exposure, which is consistent with the finding that students who had taken psychiatry courses or completed clinical rotations had lower stigma scores in univariate analysis. however, their roles as mediating variables were not directly tested in this study. Higher empathy scores were associated with lower stigma scores. Because this study employed a cross-sectional design, it is not possible to rule out reverse causality (i.e., students with lower levels of stigma being more willing to engage in empathetic interactions) or the influence of residual confounding factors.

### 4.3 Other relevant factors and educational implications

In univariate analysis, psychiatry-related coursework, clinical experience, and family history of mental illness were all associated with stigma; however, after controlling for empathy, the associations of these factors were no longer significant. This suggests that the variance they share with stigma may overlap with empathy, or that the study lacked sufficient statistical power to detect small effects of these covariates. Medical schools may still consider the following as educational entry points: Psychiatric nursing education should include a certain number of course hours, internships, and clinical practice (29). Gaining relevant theoretical knowledge before interacting with psychiatric patients in clinical settings can enhance understanding of the illness and provide a deeper sense of experience; narrative nursing education (30)—an approach proven to improve undergraduate nursing students’ mastery of psychiatric knowledge and their acceptance of psychiatric patients—as well as standardized patient simulations focused on schizophrenia care (31); and renaming schizophrenia to reduce public stigma (32). Therefore, schools can implement courses focused on empathy, incorporating formats such as scenario-based dialogues and role-playing to help students better understand mental illness and improve their empathy.

### 4.4 Limitations

First, this study employed convenience sampling at a single medical university in Shandong Province, which limits its external validity; the findings should not be generalized to vocational school or college students, other regions, or practicing nurses. Second, the cross-sectional study design does not allow for causal inferences. Third, patient-level outcome measures, such as symptoms, adherence, and perceived quality of care, were not measured. Future research should involve multicenter studies using probability sampling and, where appropriate, educational intervention trials.

## 5 Conclusion

Empathy among undergraduate nursing students is negatively correlated with public stigma toward schizophrenia. The findings of this study support the view that empathy should be regarded as an educational goal that can be cultivated through experiential learning, simulation training, and narrative-based teaching. It is recommended that relevant authorities develop strategies and implement targeted measures to foster empathy among nursing students.

## Data Availability

The raw data supporting the conclusions of this article will be made available by the authors, without undue reservation.
